# SPP1 promotes anoikis resistance and metastatic phenotypes in hepatocellular carcinoma through PI3K-sensitive signaling

**DOI:** 10.3389/fimmu.2026.1980175

**Published:** 2026-10-20

**Authors:** Xinyue Zhang, Honglong Wei, Reguang Pan, Qiming Ying, YanJin Dong, Mingfeng Xia

**Affiliations:** 1 Department of Blood Transfusion, The First Affiliated Hospital of Ningbo University, Ningbo, Zhejiang, China; 2 Department of Hepatobiliary Surgery, The First Affiliated Hospital of Shandong First Medical University & Shandong Provincial Qianfoshan Hospital, Jinan, China; 3 Department of Hepatobiliary and Pancreatic Surgery, The First Affiliated Hospital of Ningbo University, Ningbo, Zhejiang, China; 4 Department of Clinical Laboratory, Jinan Second People’s Hospital, Jinan, Shandong, China; 5 Institute of Pharmacy (Institute of Traditional Chinese Medicine (TCM) Health Industrial Technology), Shandong University of Traditional Chinese Medicine, Jinan, China

**Keywords:** alpha-fetoprotein, anoikis resistance, circulating biomarker, hepatocellular carcinoma, myeloid microenvironment, osteopontin, PI3K signaling, SPP1

## Abstract

**Background:**

Anoikis resistance enables tumor cells detached from the extracellular matrix to survive during metastatic dissemination, yet its relationship with the immune microenvironment of hepatocellular carcinoma (HCC) remains incompletely understood. Secreted phosphoprotein 1 (SPP1), which encodes osteopontin (OPN), has been implicated in HCC progression. This study investigated the role of SPP1 in anoikis resistance and metastatic phenotypes and evaluated the clinical relevance of circulating OPN in HCC.

**Methods:**

SPP1 was prioritized through integrated analyses of TCGA-LIHC data, including differential expression, survival association, intersection with anoikis-related genes, and pathway enrichment, followed by characterization of the associated tumor microenvironment. Single-cell transcriptomic analysis was performed to determine the cellular distribution of SPP1 and infer potential intercellular communication. SPP1 expression was validated in HCC tissues and cell lines. Gain- and loss-of-function experiments assessed cell viability, clonogenic growth, migration, invasion, and apoptosis under poly-HEMA suspension. The involvement of PI3K signaling was examined using LY294002. Subcutaneous xenograft and experimental pulmonary colonization models were used to evaluate *in vivo* phenotypes. Serum OPN and alpha-fetoprotein (AFP) were measured in 58 patients with HCC and 36 healthy controls.

**Results:**

SPP1 was upregulated in HCC and associated with shorter overall survival. Tumors with high SPP1 expression exhibited a myeloid- and stroma-enriched microenvironmental profile. Single-cell analysis identified prominent SPP1 expression in myeloid populations and inferred potential SPP1-mediated communication between macrophage subsets and malignant hepatocytes. Increased SPP1 expression was independently confirmed in HCC tissues and cell lines. SPP1 overexpression enhanced cell viability, clonogenic growth, migration, invasion, and resistance to suspension-induced apoptosis, whereas SPP1 silencing produced reciprocal effects. LY294002 attenuated SPP1-induced AKT activation, anoikis resistance, motility, and mesenchymal-marker expression, supporting the involvement of PI3K-sensitive signaling. Bidirectional manipulation of SPP1 produced reciprocal effects on xenograft growth and pulmonary colonization-related phenotypes. Serum OPN concentrations were elevated in HCC and were higher in patients with advanced-stage or metastatic disease. High OPN was associated with shorter overall survival (HR, 1.93; 95% CI, 1.01–3.69) and disease-free survival (HR, 2.29; 95% CI, 1.19–4.39).In the same cohort, the areas under the receiver operating characteristic curve for distinguishing patients with HCC from healthy controls were 0.819 for OPN, 0.781 for AFP, and 0.879 for their combination. These findings represent exploratory same-cohort discrimination and require validation in an independent, clinically representative at-risk population.

**Conclusion:**

SPP1 contributes to anoikis resistance and metastatic phenotypes in HCC, and these phenotypes are sensitive to PI3K inhibition. Single-cell analyses place SPP1 within a myeloid-associated tumor microenvironment, while the inferred communication signals require further experimental validation. Circulating OPN was associated with aggressive HCC features in the present cohort, and its combination with AFP warrants exploratory evaluation in independent, clinically representative at-risk populations.

## 1 Introduction

Hepatocellular carcinoma (HCC) constitutes the predominant form of primary liver cancer and remains a major cause of cancer-related mortality worldwide (1, 2). Although advances in surgical, locoregional, and systemic therapies have expanded treatment options, recurrence and metastatic dissemination continue to limit durable survival (2–4). Alpha-fetoprotein (AFP), the most widely used circulating biomarker for HCC, has suboptimal sensitivity when used alone, particularly for early-stage disease, and incompletely reflects the biological heterogeneity underlying tumor progression (4, 5). There is therefore a clinical need for complementary circulating markers that are mechanistically linked to aggressive HCC phenotypes.

Anoikis is a form of programmed cell death triggered by the loss of appropriate cell–extracellular matrix attachment and serves as an intrinsic barrier to metastatic dissemination (6). To survive vascular transit and establish secondary lesions, detached tumor cells must acquire anoikis resistance through coordinated alterations in adhesion-dependent signaling, apoptotic regulation, oxidative stress responses, and epithelial–mesenchymal plasticity (7, 8). Secreted phosphoprotein 1 (SPP1) encodes osteopontin (OPN), a secreted phosphoglycoprotein involved in extracellular matrix remodeling, inflammation, tissue repair, and cancer progression (9, 10). Through context-dependent interactions with CD44 variants and integrins, OPN can activate intracellular programs that support cell survival, migration, invasion, and treatment resistance (11, 12). These properties provide a plausible mechanistic basis through which the SPP1/OPN axis may facilitate the survival and dissemination of detached HCC cells.

The biological activity of SPP1 in HCC is embedded within a heterogeneous tumor microenvironment rather than being restricted to malignant hepatocytes. Single-cell and spatial transcriptomic studies have identified SPP1-expressing macrophage and stromal populations within hypoxic, stem-like, and immune-excluding regions of HCC (13–17). In particular, SPP1-positive macrophages and cancer-associated fibroblasts contribute to spatially organized cellular compartments associated with immune exclusion and adverse tumor states (15, 18). Fibroblast-derived SPP1 has also been shown to promote survival signaling and resistance to sorafenib and lenvatinib in malignant hepatocytes, while recurrent SPP1-centered ligand–receptor programs have been detected across spatially distinct liver tumor regions (19, 20).Collectively, these findings implicate SPP1 in myeloid–stromal niche organization and tumor-cell behavior, but do not establish that macrophage-derived SPP1 directly causes anoikis resistance in HCC cells. Accordingly, the present study treats single-cell ligand–receptor analyses as hypothesis-generating evidence and evaluates the effects of SPP1 on anoikis-related and metastatic phenotypes separately through direct perturbation in HCC cell lines.

Previous experimental studies have implicated SPP1/OPN in HCC growth, invasion, and metastasis (21–23), while clinical investigations have associated circulating OPN with tumor detection, disease burden, and prognosis and have suggested that it may complement AFP (24–26). Nevertheless, the microenvironmental, functional, and circulating-biomarker dimensions of SPP1 have largely been investigated separately. We therefore integrated bulk and single-cell transcriptomic analyses with human tissue validation, reciprocal SPP1 perturbation, time-resolved assessment of detachment-induced apoptosis, pharmacological inhibition of PI3K signaling, and *in vivo* tumor models. Circulating OPN was further evaluated in relation to tumor stage, metastatic status, survival, and its discriminatory performance alone and in combination with AFP. This study was designed to define the cellular and microenvironmental context of SPP1 expression, establish its functional contribution to anoikis-resistant and metastatic HCC phenotypes, and determine whether circulating OPN provides a measurable correlate of aggressive tumor biology.

## 2 Materials and methods

### 2.1 Bulk transcriptomic analysis

RNA-sequencing data and corresponding clinical information for The Cancer Genome Atlas Liver Hepatocellular Carcinoma cohort (TCGA-LIHC) were obtained from the Genomic Data Commons (26). The dataset comprised 371 HCC tissues and 50 non-tumor liver tissues. Differentially expressed genes were identified using an absolute log2 fold change >1 and a Benjamini–Hochberg-adjusted P<0.05. Patients were stratified according to the median SPP1 expression level for overall survival analysis. Candidate genes were prioritized by intersecting genes that were upregulated in HCC, associated with adverse survival, and included in a predefined set of 83 anoikis-related genes. Gene set enrichment analysis was performed using Hallmark and curated gene sets from the Molecular Signatures Database (27–29). ImmuneScore, StromalScore, ESTIMATEScore, and inferred tumor purity were calculated using ESTIMATE, and tumor-microenvironment cell-population signals were quantified using MCP-counter (30, 31). Associations between SPP1 expression and microenvironmental or cell-state scores were assessed using Spearman correlation and tumor-purity-adjusted partial correlation analyses.

### 2.2 Single-cell transcriptomic analysis

Single-cell count matrices and original cell annotations from GSE149614 were analyzed in Python using Scanpy and AnnData. The dataset included normal liver, primary HCC, portal vein tumor thrombus, and metastatic lymph-node specimens. Cells with at least 200 detected genes and no more than 25% mitochondrial transcripts were retained, and genes detected in fewer than three cells were excluded. The available workflow did not include computational doublet removal or explicit batch correction. Counts were normalized to 10,000 per cell and log1p-transformed. For the overview atlas, up to 120 cells per original cell type were sampled from each sample using a fixed random seed of 20260824, whereas all available myeloid cells were retained for the myeloid analysis. The overview and myeloid analyses used up to 2,500 and 2,200 highly variable genes, respectively. Principal-component analysis was performed using 40 components for the overview atlas and 35 components for the myeloid subset; the first 35 and 30 components, respectively, were used to construct neighborhood graphs, followed by UMAP visualization. Myeloid cells were clustered using the Leiden algorithm with a resolution of 0.55 and two iterations and were annotated using predefined marker modules. The SPP1-expressing macrophage module comprised SPP1, APOC1, APOE, GPNMB, LGALS3, MMP9, TREM2, and CTSB. SPP1-receptor scores were calculated in hepatocytes from tumor samples using CD44, ITGA4, ITGA5, ITGA8, ITGA9, ITGAV, ITGB1, ITGB3, ITGB5, and ITGB6. Log1p-normalized expression was standardized within each gene and averaged across the receptor genes; absent or invariant genes were excluded. Within each patient, cells at or above the 75th percentile were classified as receptor-high and those at or below the 25th percentile as receptor-low, whereas intermediate cells were excluded. Cell-state scores for anoikis resistance, PI3K–AKT–mTOR signaling, epithelial–mesenchymal transition, focal adhesion, extracellular-matrix organization, and apoptosis were calculated using the same standardized-mean approach after excluding SPP1 from the gene sets. Patient-level mean scores were compared using two-sided paired Wilcoxon tests with Benjamini–Hochberg correction across the six state comparisons. Potential ligand–receptor communication from myeloid subsets to hepatocytes was evaluated in the tumor subset using LIANA with expr_prop=0.05, min_cells=10, and n_perms=1,000, applying the CellChatDB and CellPhoneDB resources (32). Because no per-cell malignancy threshold was applied, the analyzed cells are referred to as hepatocytes from tumor samples, and all inferred communication and receptor-associated state relationships are interpreted as candidate associations rather than direct experimental evidence.

### 2.3 Patients and clinical specimens

HCC tissues, adjacent liver tissues, and serum samples were obtained from the First Affiliated Hospital of Ningbo University. SPP1 mRNA expression was measured by quantitative reverse-transcription polymerase chain reaction (qRT-PCR) in 30 paired HCC and adjacent liver tissues, comprising 16 patients with stage I–II disease and 14 with stage III–IV disease. Western blot analysis was performed using eight paired HCC and adjacent liver tissues. Immunohistochemical analysis included 60 HCC tissues and 58 adjacent liver tissues.

The serum cohort comprised 58 patients with HCC and 36 healthy controls. Among the patients with HCC, 31 had stage I–II disease and 27 had stage III–IV disease; 26 had documented metastatic disease and 32 had no documented metastasis. Blood samples were centrifuged after collection, and the separated serum was aliquoted and stored at −80 °C until analysis. For survival analyses, patients were divided into high- and low-OPN groups according to the median serum OPN concentration, with 29 patients in each group. The studies involving human participants were approved by the Ethics Committee of the First Affiliated Hospital of Ningbo University (approval/acceptance no. 2026-R261-01). All participants provided written informed consent. The study was conducted in accordance with the Declaration of Helsinki.

### 2.4 Cell culture and transfection

The human HCC cell lines SNU-387, SNU-449, HepG2, and Huh7 and the non-malignant hepatic epithelial cell line THLE-2 were examined for endogenous SPP1 expression. Based on basal SPP1 expression, SNU-387 and Huh7 cells were selected for loss- and gain-of-function experiments, respectively. Cells were maintained under the culture conditions recommended for each cell line at 37 °C in a humidified atmosphere containing 5% CO_2_. SNU-387 cells were transfected with negative-control small interfering RNA (siNC) or either of two SPP1-targeting siRNAs (siSPP1–1 and siSPP1-2; GeneChem, Shanghai, China) at a final concentration of 50 nM using Lipofectamine 3000 (Thermo Fisher Scientific; Cat. No. L3000015). Both siRNAs were initially evaluated for knockdown efficiency. Because siSPP1–2 produced the greater reduction in SPP1 expression, it was selected for subsequent functional and mechanistic experiments and is referred to as siSPP1 hereafter. Huh7 cells were transfected with an empty pcDNA3.1 vector or a full-length SPP1 expression plasmid (GeneChem) at 2 μg per well in six-well plates. Cells were harvested 48–72 h after transfection. Stable SPP1-knockdown SNU-387 cells, SPP1-overexpressing Huh7 cells, and their corresponding controls were generated for the animal experiments by selection with 2 μg/mL puromycin for 7 days. Transient siRNA transfection was used for the *in vitro* loss-of-function experiments, whereas the stable shSPP1 and shNC cell lines described in Section 2.12 were used for the *in vivo* experiments.

### 2.5 Quantitative reverse-transcription PCR

Total RNA was extracted from tissues and cells using RNAiso Plus (Takara; Cat. No. 9109). RNA (600 ng) was reverse-transcribed in a 10-μL reaction using the PrimeScript RT Reagent Kit (Takara; Cat. No. RR037A). Quantitative PCR was performed using PerfectStart Universal Green qPCR SuperMix (TransGen Biotech, Beijing, China; Cat. No. AQ631) on a ViiA 7 Real-Time PCR System (Applied Biosystems).

The amplification program comprised initial denaturation at 95 °C for 1 min, followed by 40 cycles at 95 °C for 5 s and 60 °C for 30 s. Melting-curve analysis was performed to confirm amplification specificity. Each sample was analyzed in technical triplicate. GAPDH and ACTB were used as the reference genes for tissue and cell analyses, respectively. Relative SPP1 expression was calculated using the 2^^−ΔΔCt^ method (33). Primer sequences are provided in **Supplementary Table S2**.

### 2.6 Western blot analysis

Total protein was extracted from tissues and cells using RIPA lysis buffer supplemented with protease inhibitors. Phosphatase inhibitors were additionally included when phosphorylated proteins were examined. Protein concentrations were determined using a bicinchoninic acid assay. Equal amounts of protein (30–40 μg per lane) were separated by sodium dodecyl sulfate–polyacrylamide gel electrophoresis and transferred to polyvinylidene fluoride membranes. Membranes were blocked for 1 h; 5% bovine serum albumin was used for phosphoprotein analysis.

Membranes were incubated overnight at 4 °C with primary antibodies against cellular or tissue-associated SPP1/OPN (Cell Signaling Technology [CST]; Cat. No. 8029), E-cadherin (CST; Cat. No. 3195), N-cadherin (CST; Cat. No. 13116), vimentin (CST; Cat. No. 5741), Snail (CST; Cat. No. 3879), phospho-AKT Ser473 (CST; Cat. No. 4060), AKT (CST; Cat. No. 4691), phospho-mTOR Ser2448 (CST; Cat. No. 5536), mTOR (CST; Cat. No. 2983), PTEN (CST; Cat. No. 9188), cleaved caspase-3 (CST; Cat. No. 9664), cleaved PARP (CST; Cat. No. 5625), BAX (CST; Cat. No. 2772), Bcl-2 (CST; Cat. No. 15071), or β-actin (Proteintech; Cat. No. 66009-1-Ig).After incubation with the appropriate horseradish peroxidase-conjugated secondary antibodies, immunoreactive bands were visualized using enhanced chemiluminescence. Band intensities were quantified using ImageJ (34). Target-protein abundance was normalized to β-actin, whereas phospho-AKT and phospho-mTOR were normalized to their corresponding total proteins.

### 2.7 Immunohistochemistry

Formalin-fixed, paraffin-embedded tissues were cut into 4-μm sections. The sections were deparaffinized, rehydrated, and subjected to heat-induced antigen retrieval. After endogenous peroxidase activity and nonspecific binding were blocked, the sections were incubated with primary antibodies against SPP1 (Abcam; Cat. No. ab214050; 1:200), Ki-67 (CST; Cat. No. 9449; 1:200), or cleaved caspase-3 (CST; Cat. No. 9664; 1:200).

Immunoreactivity was detected using an HRP-polymer detection system ZSGB−BIO;Cat.No.PV−9001 and 3,3′-diaminobenzidine chromogen ZSGB−BIO;Cat.No.ZLI−9018, followed by hematoxylin counterstaining. Images within each experiment were acquired using identical microscope settings. Positive staining was segmented using ImageJ, with a constant threshold applied within each comparison. SPP1 staining in human tissues was quantified as positive-staining area, whereas Ki-67 and cleaved caspase-3 staining in xenograft tissues was expressed as the percentage of positive area.

### 2.8 Cell viability and colony formation assays

Transfected cells were seeded in 96-well plates at 2,000–3,000 cells per well. At 0, 24, 48, 72, and 96 h, 10 μL of Cell Counting Kit-8 reagent (Dojindo; Cat. No. CK04) was added to each well. After incubation for 2 h, absorbance was measured at 450 nm. Five technical wells were analyzed per condition, and their mean was treated as one biological-replicate observation within each independent experiment.

For colony-formation assays, 500 cells were seeded in each well of a six-well plate and cultured for 14 days, with the medium replaced every 3–4 days. Colonies were fixed with 4% paraformaldehyde, stained with 0.5% crystal violet, and counted. All experiments were performed as three independent biological experiments.

### 2.9 Transwell migration and invasion assays

Cell migration and invasion were evaluated using Transwell inserts with 8-μm pores (Corning; Cat. No. 3422). For migration assays, 5×10^4^ cells suspended in serum-free medium were added to uncoated upper chambers. For invasion assays, the inserts were coated with 50 μL of Matrigel diluted 1:8 (Corning; Cat. No. 356234), and 2×10^5^ cells were added to each upper chamber. Medium containing 10% fetal bovine serum was placed in the lower chamber as the chemoattractant.

After incubation for 24 h for migration or 48 h for invasion, cells remaining on the upper surface of the membrane were removed. Cells that had traversed the membrane were fixed, stained with crystal violet, and counted in five randomly selected fields at×200 magnification. All assays were performed as three independent biological experiments.

### 2.10 Suspension culture and anoikis assay

Six-well plates were coated twice with 12 mg/mL poly(2-hydroxyethyl methacrylate) dissolved in 95% ethanol (poly-HEMA; Sigma-Aldrich; Cat. No. P3933) and dried to prevent cell attachment. Transfected cells were seeded at 1 × 10^5^ cells/mL and maintained under suspension conditions for 24, 48, or 72 h.

Cells were collected and stained using an Annexin V–FITC/propidium iodide apoptosis detection kit (BD Biosciences; Cat. No. 556547), followed by analysis using a BD Accuri C6 flow cytometer. The total apoptotic fraction was defined as the sum of Annexin V-positive/PI-negative and Annexin V-positive/PI-positive cells. Parallel samples collected after suspension culture were subjected to western blot analysis of cleaved caspase-3, cleaved PARP, BAX, and Bcl-2. All experiments were performed as three independent biological experiments.

### 2.11 PI3K inhibition experiments

Huh7 cells were assigned to four groups: Vector+DMSO, SPP1-OE+DMSO, Vector+LY294002, and SPP1-OE+LY294002. The PI3K inhibitor LY294002 (Selleck Chemicals; Cat. No. S1105) was administered at a final concentration of 20 μM, and an equivalent volume of DMSO was added to the vehicle-control groups.LY294002 (CAS No. 154447-36-6; 20 μM in DMSO) was added after cell attachment. Vehicle controls received an equivalent volume of DMSO. For experiments exceeding 24 h, inhibitor-containing medium was freshly prepared and replaced every 24 h. Cell viability was assessed at 0, 24, 48, 72, and 96 h; apoptosis and migration were evaluated after 24 h, invasion after 48 h, and pathway-related protein expression after 4 h of inhibitor exposure.

Cell viability, migration, and invasion were examined as described above. Suspension-induced apoptosis was measured after 48 h of culture on poly-HEMA-coated plates. The expression of phospho-AKT and total AKT, E-cadherin, N-cadherin, vimentin, Snail, and cleaved caspase-3 was evaluated by western blotting.

### 2.12 Animal experiments

Female BALB/c nude mice aged 4–6 weeks were maintained under specific pathogen-free conditions. For subcutaneous xenograft experiments, 5×10^6^ stably transfected cells suspended in 100 μL of phosphate-buffered saline were injected subcutaneously. Four groups were examined: SNU-387 shNC, SNU-387 shSPP1, Huh7 Vector, and Huh7 SPP1-OE, with six mice per group. Tumor length and width were measured every 3 days, and tumor volume was calculated as follows: Tumor volume = length × width²/2.At the experimental endpoint, mice were euthanized by gradual filling of the chamber with carbon dioxide (CO_2_) at a displacement rate of 30% of the chamber volume per minute, followed by cervical dislocation to confirm death. The tumors were then excised, weighed, photographed, and processed for histological and immunohistochemical analyses. For xenograft IHC quantification, three independent tumors per group were analyzed; multiple microscopic fields were summarized within each tumor before group-level comparison and were not treated as independent animals.

Stable SPP1 knockdown in SNU-387 cells was generated using a pLKO.1-puro lentiviral vector containing an SPP1-targeting shRNA. Lentiviral particles were produced in HEK293T cells by co-transfection with psPAX2 and pMD2.G packaging plasmids. SNU-387 cells were infected in the presence of polybrene and selected with puromycin. Knockdown was confirmed by qRT-PCR and western blotting before the animal experiments. The target sequence of shSPP1#1 was 5′-GCTGATTTGATGAACGATAAA-3′, and the non-targeting shNC sequence was 5′-TTCTCCGAACGTGTCACGT-3′.

For the experimental pulmonary colonization model, 1 × 10^6^ cells suspended in 200 μL of phosphate-buffered saline were injected through the tail vein. The SNU-387 shNC and shSPP1 groups comprised six mice each, and Huh7 Vector and Huh7 SPP1-OE cells were evaluated in parallel. At the experimental endpoint, mice were euthanized using the same carbon dioxide inhalation procedure, followed by cervical dislocation to confirm death. The lungs were then excised, photographed, fixed in formalin, embedded in paraffin, sectioned, and stained with hematoxylin and eosin. Pulmonary tumor involvement was assessed by gross examination and histopathological evaluation. Because complete animal-level quantitative measurements of pulmonary tumor burden were not available for all specimens, the gross lung images and histological sections were used as representative morphological evidence rather than as a quantitative metastatic-burden endpoint. All animal procedures were approved by the Institutional Animal Ethics Committee of Shandong University of Traditional Chinese Medicine (approval no. SDUTCM20251202002).

### 2.13 Measurement of serum OPN and AFP

Serum OPN and AFP concentrations were measured using the Human Osteopontin/OPN Quantikine ELISA Kit (R&D Systems; catalog no. DOST00; assay range, 0.312–20 ng/mL; sensitivity, 0.024 ng/mL) and the Human alpha-Fetoprotein/AFP Quantikine ELISA Kit (R&D Systems; catalog no. DAFP00; assay range, 0.312–20 ng/mL; sensitivity, 0.046 ng/mL), respectively, according to the manufacturers’ instructions. Standards and serum specimens were analyzed in duplicate. Absorbance was measured at 450 nm, and concentrations were calculated from the corresponding standard curves and expressed as pg/mL.

### 2.14 Statistical analysis

Statistical analyses were performed using GraphPad Prism 9 and R. Data are presented as the mean ± standard deviation or median with interquartile range, as appropriate. Cell-based experiments were independently repeated three times. Two-group comparisons were performed using paired or unpaired two-tailed Student’s t-tests, as appropriate, and the Mann–Whitney U-test was used for non-normally distributed data. Multiple-group and time-course comparisons were performed using one-way or two-way analysis of variance, respectively, followed by appropriate multiple-comparisons tests.

Overall survival and disease-free survival were estimated using the Kaplan–Meier method and compared using the log-rank test. Cox proportional-hazards regression was used to estimate hazard ratios and 95% confidence intervals. The discriminatory performance of serum OPN, AFP, and their combination was evaluated using receiver operating characteristic curves. The combined model was constructed using binary logistic regression with OPN and AFP as predictors, and the optimal cutoff was determined using the Youden index. AUC estimates and their 95% confidence intervals were obtained from the ROC analysis implemented in R. All tests were two-sided, and P<0.05 was considered statistically significant.

## 3 Results

### 3.1 Transcriptomic analysis prioritizes SPP1 as an anoikis-associated candidate in HCC

Analysis of the TCGA-LIHC cohort identified 1,131 upregulated and 1,130 downregulated genes in HCC tissues relative to non-tumor liver tissues, with SPP1 among the significantly upregulated genes (**Figure 1A**). Intersection of the upregulated genes, adverse-survival-associated genes, and 83 predefined anoikis-related genes identified SPP1 as the common candidate (**Figure 1B**). SPP1 expression was higher in HCC than in non-tumor liver tissues in both the entire cohort and the paired-sample analysis (both *P<0.001*; **Figure 1C**), Patients with high SPP1 expression had shorter overall survival than those with low expression (log-rank *P<0.001*; HR, 1.87; 95% CI, 1.34–2.61; **Figure 1D**).

**Figure 1:**
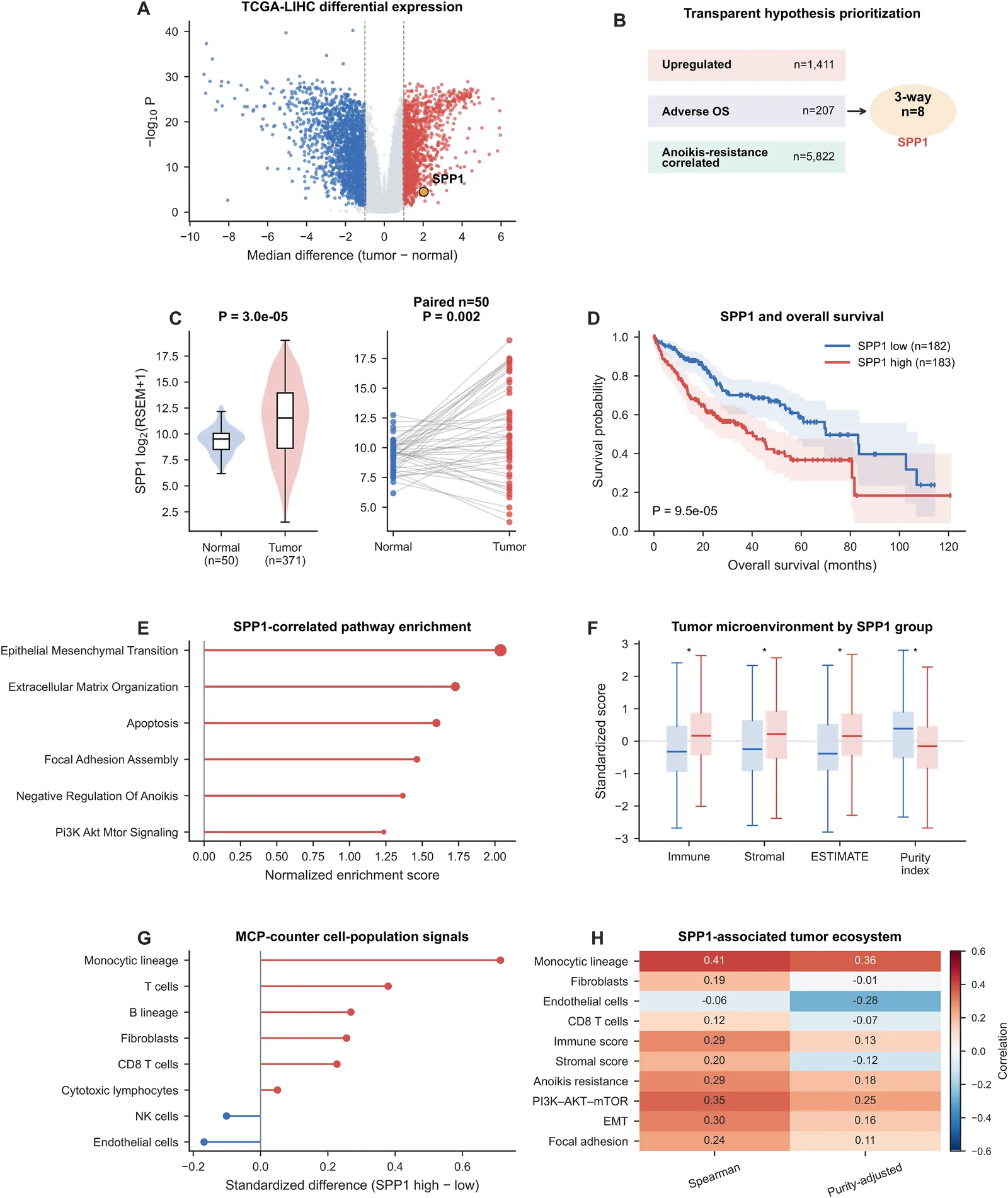
Transcriptomic prioritization identifies SPP1 within an anoikis-associated, myeloid- and stroma-enriched HCC context. **(A)** TCGA-LIHC differential expression. **(B)** Three-way prioritization of upregulated, adverse-survival-associated, and anoikis-resistance-correlated genes. **(C)** SPP1 expression in non-tumor liver and HCC, including paired samples. **(D)** Overall survival according to SPP1 expression. **(E)** SPP1-correlated pathway enrichment. **(F)** Tumor-microenvironment scores by SPP1 group. **(G)** MCP-counter cell-population signals. **(H)** Spearman and tumor-purity-adjusted correlations between SPP1 and ecosystem/state scores. MCP, Microenvironment Cell Populations; EMT, epithelial–mesenchymal transition.

Gene set enrichment analysis associated high SPP1 expression with epithelial–mesenchymal transition (NES = 2.14, FDR *q=0.001*), PI3K–AKT–mTOR signaling, focal adhesion, cell–matrix interaction, migration, hypoxia, and extracellular-matrix–receptor interaction (**Figure 1E**). Tumors with high SPP1 expression also exhibited higher immune, stromal, and composite ESTIMATE scores and lower inferred tumor purity (**Figure 1F**). MCP-counter analysis indicated stronger monocytic-lineage and fibroblast signals in the SPP1-high group (**Figure 1G**). SPP1 expression correlated positively with myeloid, stromal, and aggressive tumor-state scores, including anoikis resistance, PI3K–AKT–mTOR signaling, epithelial–mesenchymal transition, and focal adhesion. These associations remained directionally consistent after adjustment for inferred tumor purity (**Figure 1H**). Collectively, these analyses prioritized SPP1 as an anoikis-associated candidate within a myeloid- and stroma-enriched HCC context.

### 3.2 Single-cell analysis identifies an SPP1-expressing macrophage compartment associated with hepatocyte states

Single-cell analysis of GSE149614 included 71,915 cells from normal liver, primary HCC, portal vein tumor thrombus, and metastatic lymph-node specimens (**Figure 2A**). Unsupervised clustering and canonical lineage-marker expression identified T/NK cells, hepatocytes, myeloid cells, B cells, endothelial cells, and fibroblasts (**Figures 2B, C**). SPP1 expression was concentrated predominantly within the myeloid compartment (**Figure 2D**).

**Figure 2:**
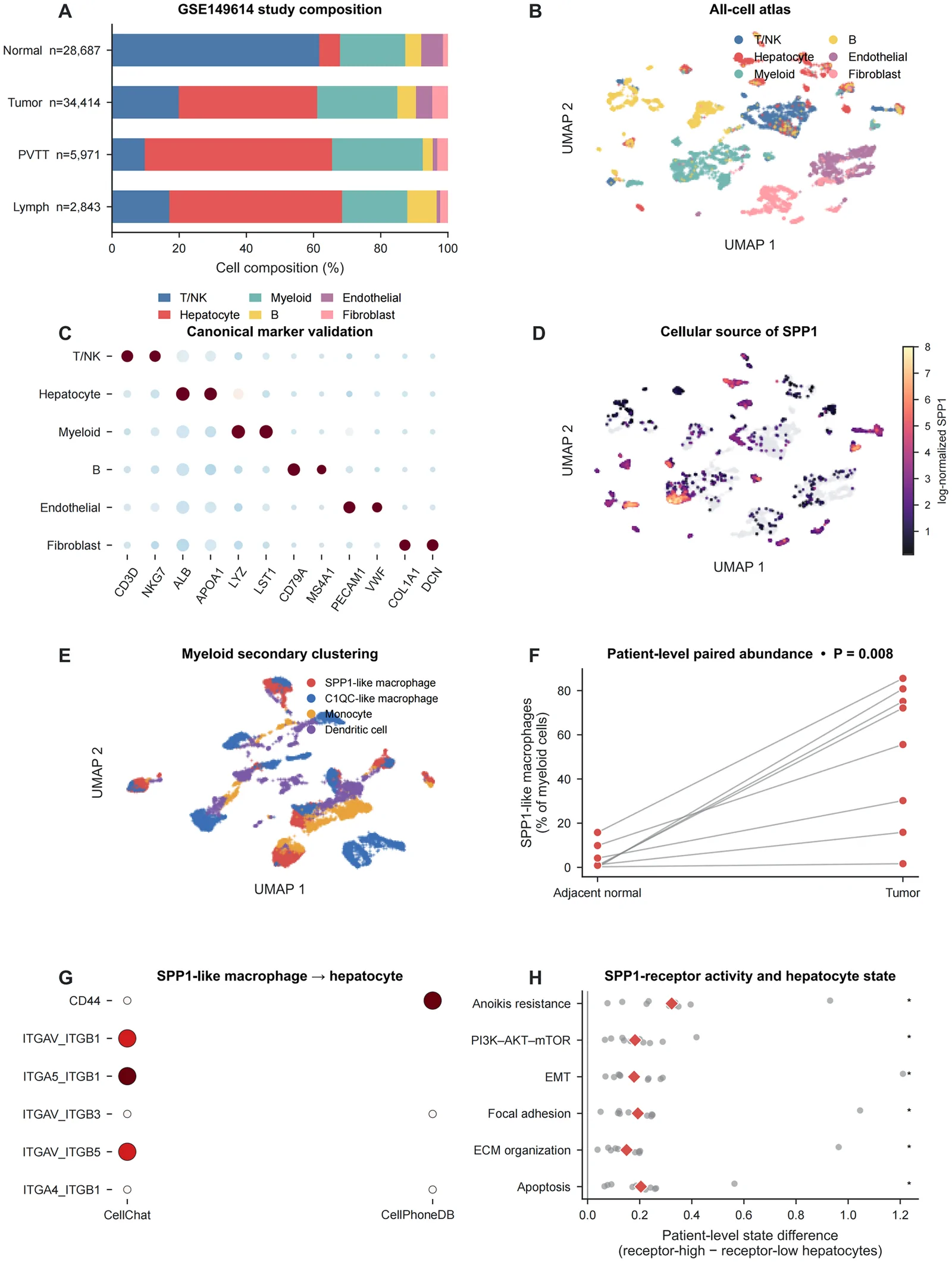
Single-cell profiling defines the cellular distribution and inferred intercellular signaling context of SPP1 in HCC. **(A)** Cellular composition across normal liver, primary HCC, portal vein tumor thrombus, and metastatic lymph-node samples in GSE149614. **(B)** All-cell UMAP atlas. **(C)** Canonical marker validation. **(D)** Cellular distribution of SPP1. **(E)** Secondary clustering of myeloid cells. **(F)** Patient-level paired abundance of SPP1-expressing macrophages. **(G)** Putative SPP1-expressing macrophage-to-hepatocyte signaling inferred using CellChat and CellPhoneDB. **(H)** Differences in hepatocyte state scores according to SPP1-receptor activity. UMAP distributions and cluster annotations are descriptive at the cell or cluster level, whereas the paired abundance comparison in panel F was performed at the patient level. The communication analysis and receptor-associated hepatocyte state comparisons represent inferred associations and do not constitute direct experimental validation. PVTT, portal vein tumor thrombus; EMT, epithelial–mesenchymal transition; ECM, extracellular matrix.

Re-clustering of myeloid cells resolved SPP1-expressing macrophages, C1QC-expressing macrophages, monocytes, and dendritic cells (**Figure 2E**). The proportion of SPP1-expressing macrophages within the myeloid compartment was compared at the patient level and was higher in tumor tissues than in paired adjacent non-tumor tissues (P = 0.008; **Figure 2F**). LIANA-based ligand–receptor analysis, supported by CellChatDB and CellPhoneDB resources, inferred candidate communication from SPP1-expressing macrophages to hepatocytes through CD44- and integrin-containing receptor complexes (**Figure 2G**). These inferred interactions were not directly validated experimentally. Compared with receptor-low hepatocytes, receptor-high hepatocytes exhibited higher patient-level mean scores for anoikis resistance, PI3K–AKT–mTOR signaling, epithelial–mesenchymal transition, focal adhesion, and extracellular-matrix organization, together with a difference in the apoptosis-related score (**Figure 2H**). These analyses identify a computationally inferred communication context associated with hepatocyte transcriptional states but do not establish a causal effect of macrophage-derived SPP1 on hepatocytes. The functional experiments described below directly manipulated SPP1 in HCC cells and independently assessed the resulting cellular phenotypes.

### 3.3 SPP1 is upregulated in independent HCC tissues and cell lines

In an independent cohort of 30 paired specimens, SPP1 mRNA expression was significantly higher in HCC tissues than in adjacent liver tissues (*P<0.01*; **Figure 3A**). SPP1 mRNA levels were also higher in stage III–IV tumors than in stage I–II tumors (*P=0.022*; **Figure 3B**). Consistent with the transcript-level findings, western blot analysis showed increased SPP1 protein abundance in HCC tissues relative to adjacent tissues (*P < 0.01*; **Figure 3C**).

**Figure 3:**
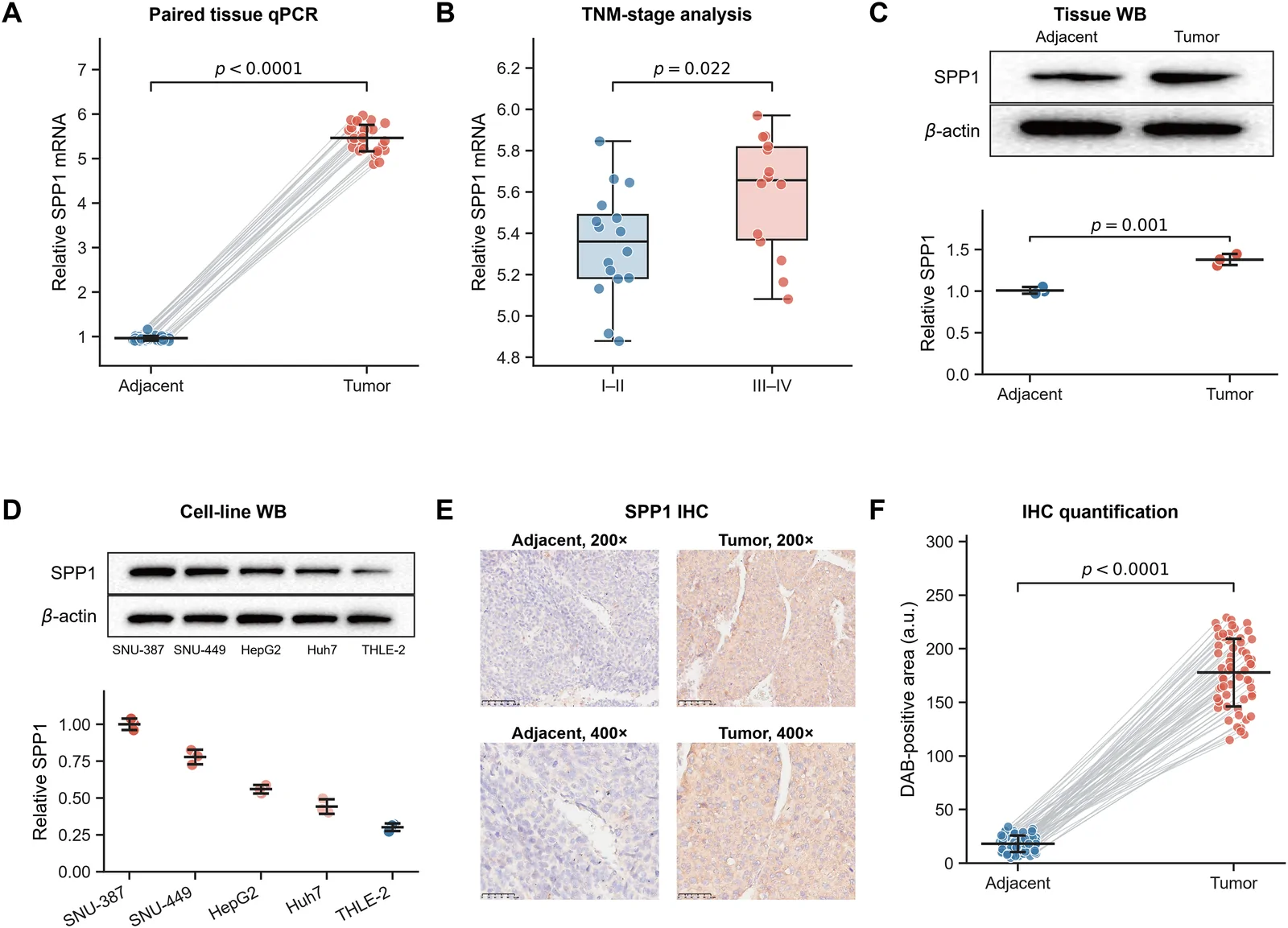
SPP1 is upregulated in HCC tissues and cell lines. **(A)** qRT-PCR in 30 matched HCC and adjacent tissues; each point represents one independent tissue specimen. **(B)** SPP1 mRNA by TNM stage (I–II, n=16; III–IV, n=14); each point represents a tumor specimen from one patient. **(C)** Representative tissue western blot and densitometric quantification from three matched tissue pairs analyzed in the same batch; these points are clinical specimens, not cell-based biological replicates. **(D)** Representative SPP1 western blot across HCC lines and THLE-2; densitometric values derive from three independent biological experiments (n=3). **(E)** Representative SPP1 IHC at 200× and 400×. **(F)** Area-based IHC quantification for matched specimens; the complete IHC set contained 60 HCC and 58 adjacent tissues. Linked lines connect matched tissues from the same patient, and each point represents one independent tissue specimen. Exact p values are shown. WB, western blot; IHC, immunohistochemistry; DAB, 3,3′-diaminobenzidine.

SPP1 protein abundance varied across the examined cell lines, with the highest level detected in SNU-387 cells, followed by SNU-449, HepG2, and Huh7 cells; THLE-2 cells exhibited the lowest level (**Figure 3D**). This expression pattern supported the use of SNU-387 cells for loss-of-function experiments and Huh7 cells for gain-of-function experiments. Immunohistochemistry revealed more extensive SPP1 staining in HCC tissues than in adjacent liver tissues, and quantitative image analysis confirmed this difference (*P<0.01*; **Figures 3E, F**). Thus, increased SPP1 expression in HCC was consistently detected at the transcript, protein, and histological levels.

### 3.4 Reciprocal SPP1 perturbation modulates HCC cell growth and motility

SPP1 overexpression in Huh7 cells and SPP1 silencing in SNU-387 cells were confirmed before functional assessment (**Figures 4A, B**). Both siSPP1–1 and siSPP1–2 reduced SPP1 abundance relative to siNC, with siSPP1–2 producing the greater reduction. Therefore, siSPP1–2 was used in subsequent loss-of-function experiments.

**Figure 4:**
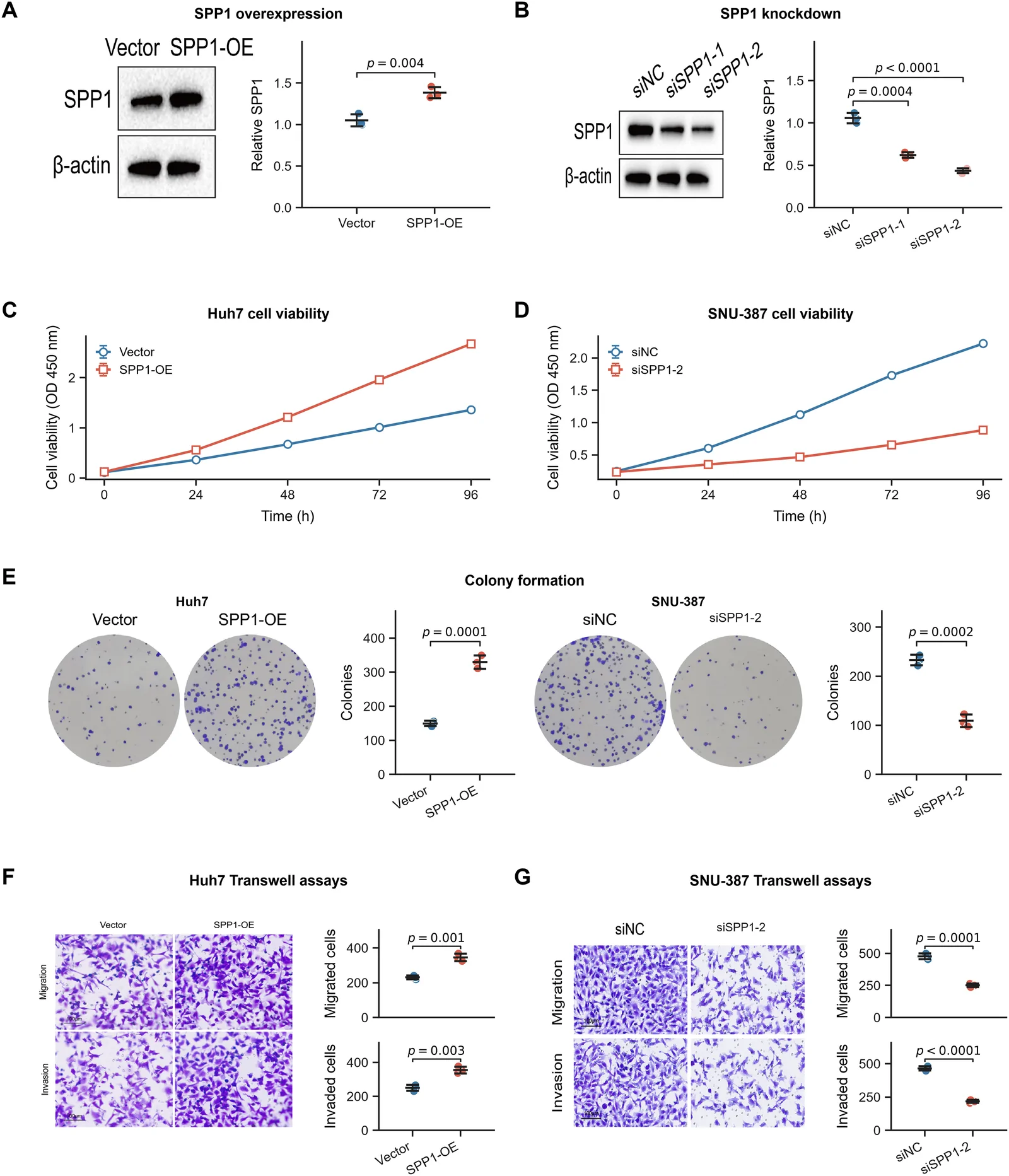
SPP1 promotes HCC cell viability, clonogenic growth, migration, and invasion. **(A)** Western-blot validation of SPP1 overexpression in Huh7 cells. **(B)** Screening of siSPP1–1 and siSPP1–2 in SNU-387 cells. siSPP1–2 produced the greater knockdown and was selected for subsequent experiments. **(C, D)** Huh7 and SNU-387 CCK-8 trajectories. **(E)** Representative colony-formation images and quantification. **(F, G)** Representative migration and invasion images and quantification for Huh7 and SNU-387 cells. All cell-based experiments were performed as three independent biological experiments (n=3). For CCK-8 assays, the mean of five technical wells within each experiment constituted one biological-replicate observation. Points denote independent biological experiments; bars/lines show the mean and dispersion as plotted. Scale bars and p values are displayed in the panels. OE, overexpression; siNC, negative-control siRNA.

SPP1 overexpression increased the CCK-8 signal in Huh7 cells over the 96-h observation period, whereas SPP1 silencing reduced the corresponding signal in SNU-387 cells (**Figures 4C, D**). The colony-formation assay yielded consistent results. Consistently, SPP1 overexpression increased migration and invasion in Huh7 cells, whereas SPP1 silencing reduced these capacities in SNU-387 cells (all P<0.01; **Figures 4F, G**). SPP1 overexpression increased colony formation in Huh7 cells, whereas SPP1 silencing reduced colony formation in SNU-387 cells (both *P<0.01*; **Figure 4E**).

### 3.5 SPP1 promotes resistance to detachment-induced apoptosis

During 24–72 h of suspension culture, the apoptotic fraction increased progressively in vector-transfected Huh7 cells. SPP1 overexpression reduced apoptosis throughout the observation period. Conversely, SPP1 silencing increased suspension-induced apoptosis in SNU-387 cells over the same time course (**Supplementary Figures S1A, B**).

The changes in apoptosis-associated proteins were consistent with the flow-cytometric findings. After 48 h of suspension culture, SPP1 overexpression decreased cleaved caspase-3, cleaved PARP, and BAX and increased Bcl-2 in Huh7 cells. SPP1 silencing produced the reciprocal pattern in SNU-387 cells (**Supplementary Figures S1C, D**). These time-resolved and molecular findings demonstrate that SPP1 shifts the apoptotic response of HCC cells to loss of matrix attachment toward survival.

### 3.6 PI3K inhibition attenuates SPP1-induced anoikis resistance and motility

The contribution of PI3K signaling to the SPP1-responsive phenotype was examined using a four-group pharmacological intervention. Under vehicle treatment, SPP1 overexpression increased Huh7 cell viability, whereas LY294002 attenuated this effect (**Figure 5A**). Following 48 h of suspension culture, SPP1 overexpression reduced apoptosis, and LY294002 diminished this protective response (**Figure 5B**). LY294002 also attenuated the SPP1-associated increases in migration and invasion (**Figure 5C**).

**Figure 5:**
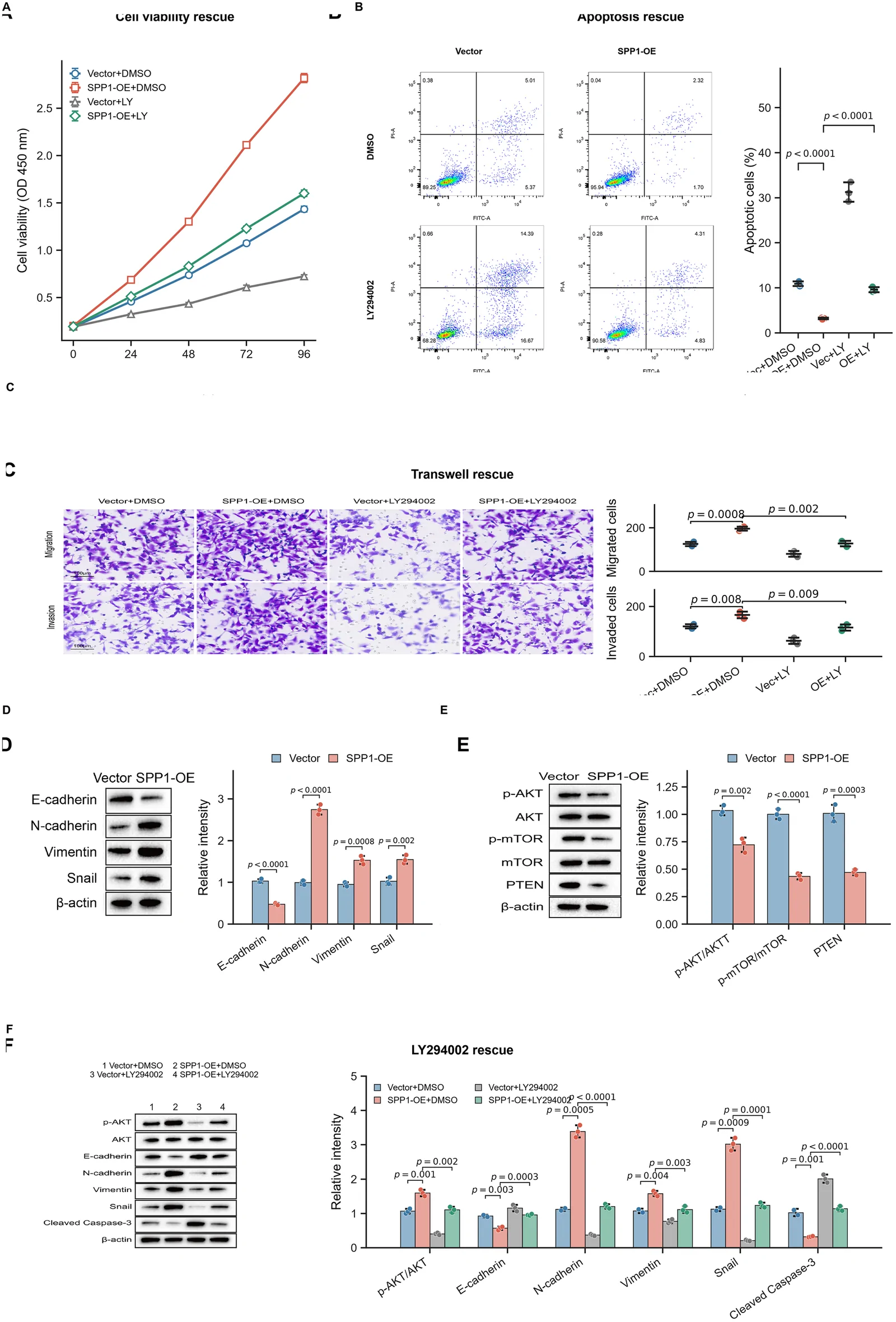
PI3K inhibition attenuates SPP1-responsive viability, anoikis resistance, motility, and EMT-marker changes. Huh7 cells were assigned to Vector+DMSO, SPP1-OE+DMSO, Vector+LY294002, and SPP1-OE+LY294002; LY294002 was used at 20 μM. **(A)** CCK-8 analysis after four-group pharmacological intervention. **(B)** Annexin V–FITC/PI analysis after 48 h of poly-HEMA suspension and quantification of total apoptotic cells. **(C)** Transwell migration and invasion. **(D)** EMT-marker immunoblotting after SPP1 overexpression. **(E)** Two-group PI3K/AKT-mTOR/PTEN immunoblotting. **(F)** Four-group immunoblotting for p-AKT/AKT, EMT markers, and cleaved caspase-3 after LY294002 rescue. All cell-based experiments were performed as three independent biological experiments (n=3). Technical replicates were averaged within each experiment before analysis. P values are displayed in the panels. EMT, epithelial–mesenchymal transition.

SPP1 overexpression decreased E-cadherin and increased N-cadherin, vimentin, and Snail expression in the EMT immunoblot (**Figure 5D**). A separate two-group immunoblot examined p-AKT, AKT, p-mTOR, mTOR, and PTEN following SPP1 overexpression (**Figure 5E**). In the four-group LY294002 experiment, SPP1 overexpression increased the p-AKT/AKT ratio, whereas LY294002 attenuated this increase and the associated changes in EMT markers and cleaved caspase-3 (**Figure 5F**). Together, these findings support an association between the SPP1-responsive phenotypes and PI3K-sensitive signaling; they do not establish a direct SPP1-to-PI3K mechanism.

### 3.7 Reciprocal SPP1 perturbation modulates xenograft growth and pulmonary colonization-related phenotypes

In the SNU-387 xenograft model, SPP1 silencing reduced tumor growth and terminal tumor weight relative to the shNC control (*P<0.01*; **Figure 6A**). The gain-of-function experiment produced the reciprocal response: SPP1 overexpression accelerated Huh7 xenograft growth and increased terminal tumor weight relative to the vector control (*P<0.01*; **Figure 6B**). Gross images of the excised tumors were consistent with the differences in tumor volume and weight (**Figure 6C**).

**Figure 6:**
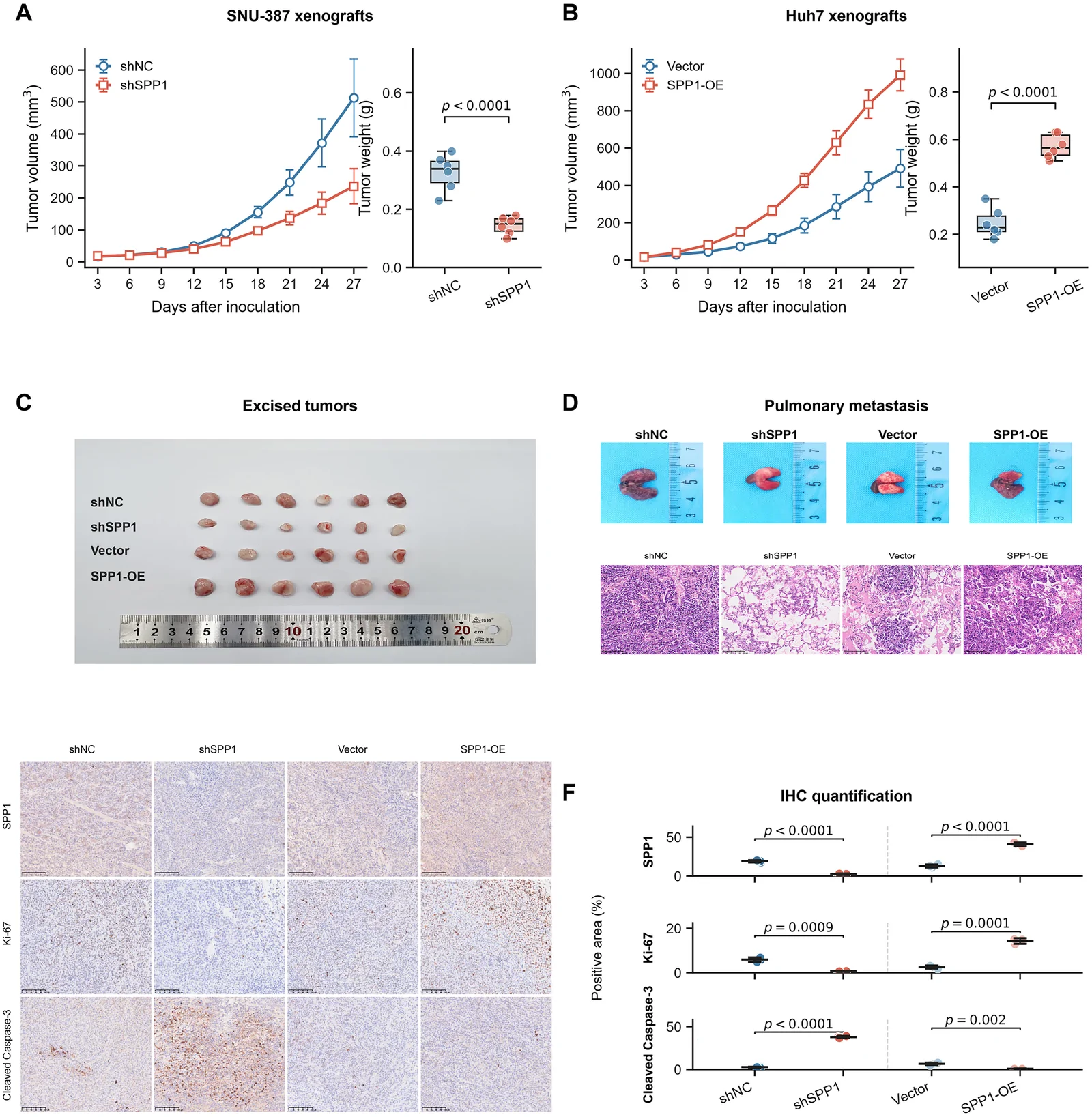
SPP1 perturbation modulates xenograft growth and pulmonary colonization-related phenotypes *in vivo*. **(A)** SNU-387 shNC and shSPP1 xenograft volumes and terminal weights (n=6 per group). **(B)** Huh7 Vector and SPP1-OE xenograft volumes and terminal weights (n=6 per group). **(C)** Excised tumors from the four xenograft groups. **(D)** Representative gross lung images and hematoxylin–eosin sections from the tail-vein pulmonary colonization model; these images are presented as qualitative morphological evidence and were not used as an animal-level quantitative metastatic-burden endpoint. **(E)** Representative xenograft IHC images for SPP1, Ki-67, and cleaved caspase-3. **(F)** Positive-area quantification based on three independent tumors per group; microscopic fields were summarized within each tumor before group comparison. P values and scale bars are displayed. H&E, hematoxylin and eosin; IHC, immunohistochemistry.

Immunohistochemical analysis showed that SPP1-silenced xenografts exhibited reduced SPP1 and Ki-67 staining and increased cleaved caspase-3 staining. SPP1-overexpressing xenografts displayed the opposite pattern (**Figures 6E, F**). Quantification was based on three independent tumors per group; multiple microscopic fields were summarized within each tumor before group-level comparison and were not treated as independent animals. In the experimental pulmonary colonization model, representative gross lung specimens and hematoxylin–eosin-stained sections showed directionally consistent differences in pulmonary tumor involvement following SPP1 silencing or overexpression (**Figure 6D**). Because complete animal-level quantitative measurements of pulmonary tumor burden were not available for all specimens, these images are presented as representative morphological evidence rather than as a quantitative metastatic-burden endpoint.

### 3.8 Circulating OPN is associated with aggressive HCC features and shows exploratory same-cohort discrimination

Serum OPN concentrations were higher in the 58 patients with HCC than in the 36 healthy controls (*P<0.001*; **Figure 7A**). Serum AFP concentrations were also higher in patients with HCC (*P<0.001*; **Figure 7B**). Within the HCC cohort, OPN concentrations were higher in patients with stage III–IV disease than in those with stage I–II disease (*P=0.033*; **Figure 7C**) and in patients with documented metastasis than in those without documented metastasis (*P=0.014*; **Figure 7D**).

**Figure 7:**
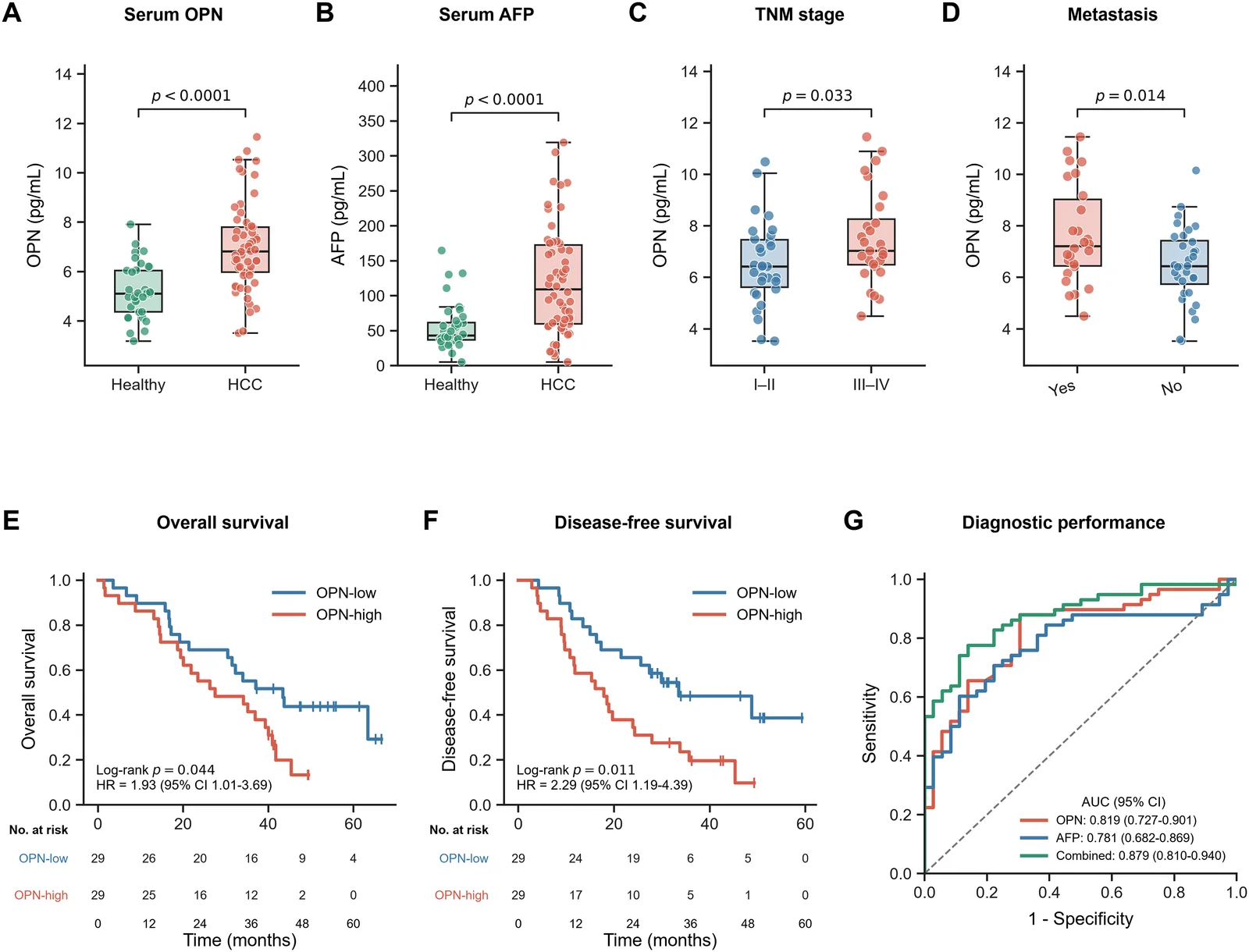
Circulating OPN is associated with HCC burden, survival, and exploratory HCC-versus-healthy-control discrimination. **(A, B)** Serum OPN and AFP in healthy controls (n=36) and patients with HCC (n=58). **(C)** OPN by TNM stage (I–II, n=31; III–IV, n=27). **(D)** OPN by metastasis status (present, n=26; absent, n=32). **(E, F)** OS and DFS after median dichotomization (n=29 per group), with numbers at risk. OS: log-rank p=0.044; HR = 1.93 (95% CI 1.01–3.69). DFS: log-rank p=0.011; HR = 2.29 (95% CI 1.19–4.39). **(G)** ROC curves for OPN, AFP, and their same-cohort logistic combination. AUCs were 0.819 (95% CI 0.727–0.901), 0.781 (0.682–0.869), and 0.879 (0.810–0.940), respectively. Concentrations are pg/mL. OPN, osteopontin; AFP, alpha-fetoprotein; OS, overall survival; DFS, disease-free survival; ROC, receiver operating characteristic; AUC, area under the curve.

The clinicopathological characteristics of the serum cohort according to the median OPN concentration are summarized (**Table 1**). Patients in the high-OPN group had numerically higher proportions of stage III–IV disease (58.6% vs. 34.5%) and metastatic disease (58.6% vs. 31.0%) than those in the low-OPN group, although these differences did not reach statistical significance (*P=0.113* and *P=0.064*, respectively). Serum AFP concentrations were comparable between the two groups (**Table 1**) ( P=0.208).After stratification at the median serum OPN concentration, patients in the high-OPN group had shorter overall survival and disease-free survival than those in the low-OPN group (log-rank *P=0.044* and *P=0.011*, respectively; **Figures 7E, F**). In univariable Cox regression analyses, high OPN was associated with shorter overall survival (HR, 1.93; 95% CI, 1.01–3.69; P = 0.048) and disease-free survival (HR, 2.29; 95% CI, 1.19–4.39; *P=0.013*) (**Table 2**).

**Table 1:** Characteristics of the 58-patient serum HCC cohort according to median OPN level.

Characteristic	Overall (n=58)	Low OPN (n=29)	High OPN (n=29)	P value
TNM stage, n (%)				0.113
I–II	31 (53.4)	19 (65.5)	12 (41.4)	
III–IV	27 (46.6)	10 (34.5)	17 (58.6)	
Metastasis, n (%)				0.064
Absent	32 (55.2)	20 (69.0)	12 (41.4)	
Present	26 (44.8)	9 (31.0)	17 (58.6)	
Serum OPN, pg/mL	6.8 [6.0–7.8]	6.0 [5.3–6.4]	7.8 [7.3–9.2]	—
Serum AFP, pg/mL	109.3 [59.7–172.7]	85.5 [56.1–176.1]	133.5 [70.0–165.9]	0.208

**Table 2:** Univariable Cox proportional-hazards analyses in the serum HCC cohort.

Outcome and variable	HR (95% CI)	P value
Overall survival		
High vs low OPN	1.926 (1.006–3.687)	0.048
TNM III–IV vs I–II	1.583 (0.846–2.962)	0.150
Metastasis present vs absent	1.410 (0.752–2.647)	0.284
Disease-free survival		
High vs low OPN	2.288 (1.192–4.394)	0.013
TNM III–IV vs I–II	1.149 (0.608–2.173)	0.668
Metastasis present vs absent	1.397 (0.744–2.621)	0.298

Separate univariable Cox models were fitted for each variable. HR, hazard ratio; CI, confidence interval; OPN, osteopontin.

In the same cohort, OPN yielded an area under the receiver operating characteristic curve of 0.819 (95% CI, 0.727–0.901), whereas AFP yielded an area of 0.781 (95% CI, 0.682–0.869). The logistic combination of OPN and AFP yielded an AUC of 0.879 (95% CI, 0.810–0.940), with 77.6% sensitivity and 86.1% specificity at the selected threshold (**Figure 7G**, **Table 3**). These estimates represent exploratory same-cohort discrimination between patients with HCC and healthy controls and should not be interpreted as externally validated diagnostic performance. Independent validation in clinically representative at-risk populations is required.

**Table 3:** Exploratory discrimination between HCC and healthy controls.

Biomarker	AUC	95% CI	Cutoff	Sensitivity	Specificity	Youden index	PPV	NPV
OPN	0.819	0.727–0.901	5.28 pg/mL	87.9%	69.4%	0.574	82.3%	78.1%
AFP	0.781	0.682–0.869	85.49 pg/mL	60.3%	88.9%	0.492	89.7%	58.2%
OPN+AFP	0.879	0.810–0.940	0.617¹	77.6%	86.1%	0.637	90.0%	70.5%

¹Predicted-probability cutoff from the same-cohort logistic model. Values describe discrimination in the development cohort. AUC, area under the curve; PPV, positive predictive value; NPV, negative predictive value.

## 4 Discussion

This study integrates bulk and single-cell transcriptomic analyses with functional perturbation, *in vivo* models, and circulating biomarker assessment to characterize the SPP1/OPN axis in HCC. High SPP1 expression was associated with an anoikis-related transcriptional program and a myeloid- and stroma-enriched tumor microenvironment. Single-cell analysis localized prominent SPP1 expression to macrophage populations and identified putative communication with hepatocytes. These cell–cell communication results were computationally inferred and should be interpreted as a microenvironmental context rather than direct evidence of macrophage-derived SPP1 activity in HCC cells. In separate functional experiments, reciprocal manipulation of SPP1 in HCC cell lines altered detachment-induced apoptosis, cell growth, motility, xenograft growth, and pulmonary colonization-related phenotypes. These complementary findings were accompanied by associations between circulating OPN and advanced stage, metastatic status, and adverse survival outcomes.

The microenvironmental context of SPP1 is particularly relevant to the formation and remodeling of immunological niches in HCC. Previous single-cell and spatial studies have identified SPP1-positive macrophages and fibroblasts within hypoxic, stem-like, and immune-excluding regions of liver tumors (13–21). SPP1-positive macrophages can participate in spatial barriers associated with immune exclusion, whereas fibroblast-derived SPP1 has been implicated in survival signaling and resistance to sorafenib and lenvatinib in malignant hepatocytes (16–20). Consistent with these observations, the present bulk transcriptomic analysis associated SPP1 with monocytic-lineage and stromal signals, and the single-cell analysis identified an expanded SPP1-expressing macrophage compartment in tumor tissue. The inferred SPP1–CD44 and SPP1–integrin interactions were associated with hepatocyte states characterized by increased anoikis-resistance, PI3K–AKT–mTOR, epithelial–mesenchymal transition, focal-adhesion, and extracellular-matrix scores. These findings provide a computationally inferred link between an SPP1-rich immune context and hepatocyte transcriptional states, whereas the direct effects of SPP1 perturbation on anoikis resistance and metastatic phenotypes were established separately in HCC cell and animal models.

Anoikis resistance is required for detached tumor cells to survive vascular transit and establish secondary lesions. Previous studies have implicated SPP1/OPN in HCC growth, invasion, metastasis, and anoikis-related transcriptional programs (22–25). The present study provides reciprocal functional evidence across two cellular backgrounds. SPP1 overexpression reduced suspension-induced apoptosis, whereas SPP1 silencing accelerated this response over a 24–72-h detachment trajectory. Concordant changes in cleaved caspase-3, cleaved PARP, BAX, and Bcl-2 supported the flow-cytometric findings. SPP1 also altered E-cadherin, N-cadherin, vimentin, and Snail expression. These results indicate that the SPP1-responsive phenotype involves both apoptotic regulation and epithelial–mesenchymal plasticity, two biologically related but distinct processes that facilitate survival and motility after loss of matrix attachment.

PI3K-sensitive signaling was associated with the SPP1-responsive phenotype. PI3K–AKT signaling integrates extracellular ligand, adhesion, metabolic, and apoptotic inputs and is frequently engaged during malignant progression (35, 36). OPN has previously been linked to PI3K–AKT-dependent survival, epithelial–mesenchymal transition, and metastatic behavior in HCC (33, 34, 37). In the present four-group experiment, LY294002 attenuated SPP1-associated AKT phosphorylation, cell viability, migration, invasion, resistance to suspension-induced apoptosis, and changes in epithelial–mesenchymal transition markers. The convergence of these effects across molecular and functional endpoints supports a PI3K-sensitive component of the SPP1-responsive phenotype but does not establish a direct SPP1-to-PI3K signaling mechanism. In the context of the single-cell findings, CD44 and integrin complexes represent plausible candidate mediators, although their individual contributions remain to be established experimentally.

The *in vivo* and clinical observations provide complementary evidence for the biological relevance of the SPP1-responsive phenotype. Bidirectional SPP1 manipulation produced reciprocal effects on xenograft growth, tumor weight, Ki-67 staining, and cleaved caspase-3 staining. The pulmonary model showed directionally consistent differences in lung involvement, providing morphological support for a colonization-related phenotype. Circulating OPN was elevated in HCC and was higher in patients with advanced-stage or metastatic disease. Higher serum OPN was also associated with shorter overall and disease-free survival in univariable analyses. The combination of OPN and AFP yielded greater discrimination than either marker alone within the same cohort; however, this exploratory performance may be optimistic because the model was developed and evaluated in the same sample. These findings support further evaluation of circulating OPN alongside AFP rather than its replacement, with validation in independent and clinically representative at-risk populations (38–40).

Several limitations should be considered. The clinical findings were derived from a single-center cohort and require validation in independent populations, particularly in patients undergoing HCC surveillance because of cirrhosis or chronic liver disease. The survival analyses were limited to univariable Cox regression, and the observed associations between serum OPN and survival were not adjusted for potential confounding factors, including tumor stage. These findings therefore do not establish serum OPN as an independent prognostic factor. The cell-communication analyses were computationally inferred, and receptor-specific experiments will be required to establish the relative contributions of CD44 and individual integrin complexes. In addition, the pulmonary colonization findings were based on representative morphological observations rather than animal-level quantitative analysis. These considerations define priorities for further clinical and mechanistic investigation without altering the internal consistency of the present findings. A working model integrating the myeloid-associated SPP1 context, inferred intercellular communication, PI3K-sensitive tumor-cell phenotypes, and circulating OPN findings is presented in **Figure 8**.

**Figure 8:**
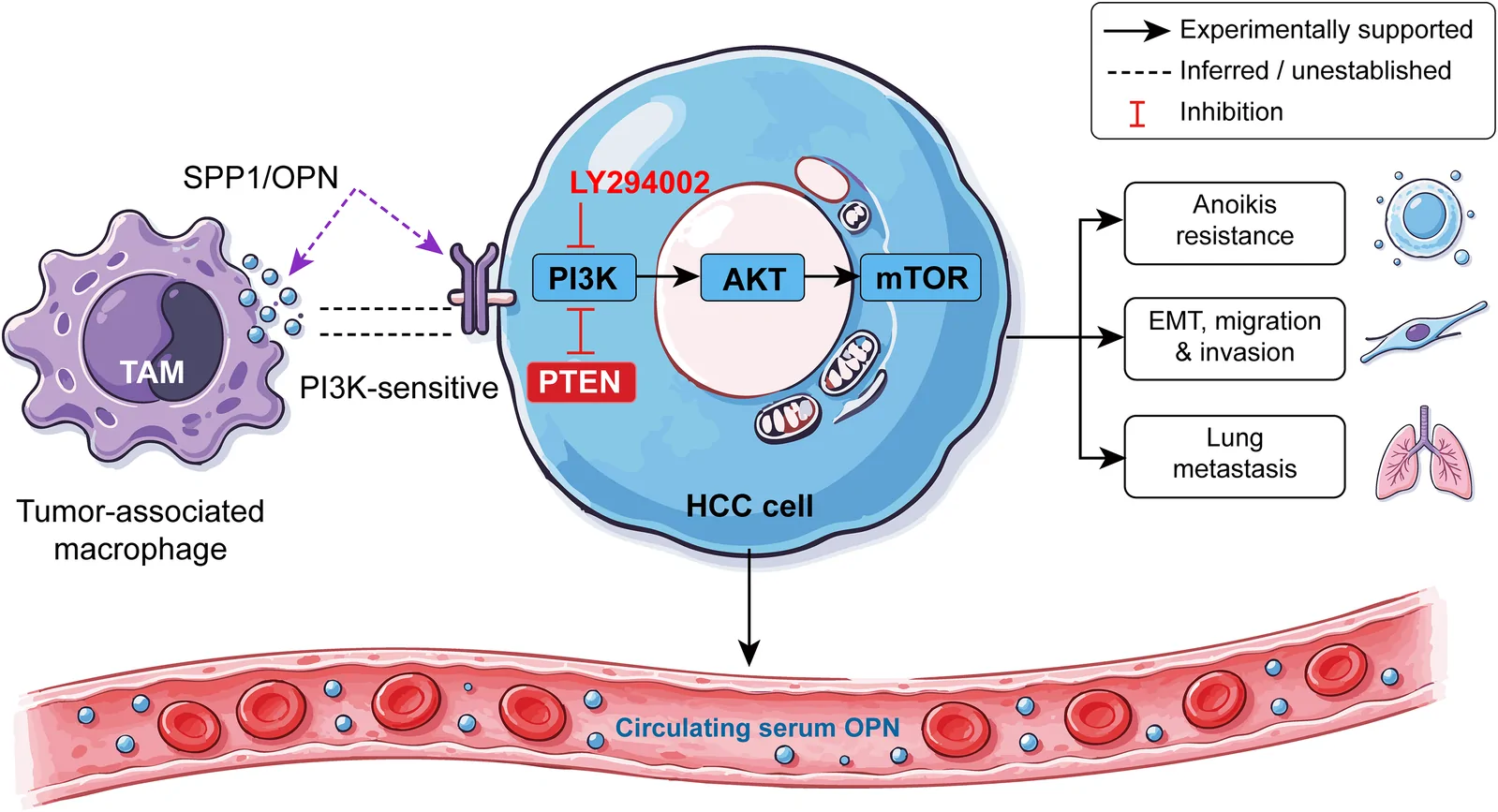
Proposed working model of SPP1/OPN-associated signaling in HCC. SPP1/OPN expression in tumor-associated macrophages and circulating OPN are integrated with the PI3K–AKT–mTOR-associated phenotype observed in HCC cells. Solid arrows indicate experimentally supported relationships, whereas dashed arrows indicate inferred or unestablished interactions. LY294002-sensitive signaling is associated with anoikis resistance, epithelial–mesenchymal transition, migration, invasion, and pulmonary colonization-related phenotypes. The direct contribution of individual receptors and the macrophage-to-tumor-cell signaling route requires further experimental validation.

In conclusion, this study provides evidence that SPP1 contributes to anoikis resistance and metastatic phenotypes in HCC and that these phenotypes are sensitive to PI3K inhibition. Single-cell analyses place SPP1 within a myeloid-associated tumor microenvironment and identify putative communication with hepatocytes, but these inferred interactions do not establish a direct causal mechanism. Circulating OPN was associated with advanced-stage disease, metastatic status, and adverse survival outcomes in the present cohort. Its combination with AFP showed exploratory same-cohort discrimination and requires validation in independent, clinically representative at-risk populations.

## Data Availability

The original contributions presented in the study are included in the article/**Supplementary Material**. Further inquiries can be directed to the corresponding authors.

## Supplementary material

Supplementary Figure 1 SPP1 shifts the time-dependent anoikis response in HCC cells. **(A)** Annexin V–FITC/PI analysis of Vector and SPP1-OE Huh7 cells after 24, 48, and 72 h in poly-HEMA suspension. **(B)** Corresponding analysis of siNC and siSPP1–2 SNU-387 cells. Total apoptotic cells are Annexin V-positive populations. **(C, D)** Western blots and densitometric values for cleaved caspase-3, cleaved PARP, Bcl-2, and BAX after suspension culture. All cell-based experiments were performed as three independent biological experiments (n=3). Technical replicates were averaged within each experiment before analysis, and points denote independent biological experiments. P values are shown in the panels.
