# Supplementary material for: SPP1 promotes anoikis resistance and metastatic phenotypes in hepatocellular carcinoma through PI3K-sensitive signaling
Source: Front Immunol. 2026 Oct 20;17:1980175. doi: 10.3389/fimmu.2026.1980175 (PMC13727754; doi:10.3389/fimmu.2026.1980175)
Supplement: Supplementary file 1 [file Table1.docx]

**Supplementary Table S1. Clinical sample sets used across analytical modules**

The sample sets were analytically distinct unless explicitly stated. AFP, alpha-fetoprotein; HCC, hepatocellular carcinoma; IHC, immunohistochemistry; OPN, osteopontin; qRT-PCR, quantitative reverse-transcription polymerase chain reaction.

| **Module** | **Clinical sample set** | **Participants** | **Specimens** | **Analytical role** |
| --- | --- | --- | --- | --- |
| qRT-PCR | 30 matched HCC and adjacent tissues | 30 patients | 60 tissues | Paired SPP1 mRNA validation |
| IHC | 60 HCC and 58 adjacent tissues | See specimen counts | 118 tissues | Histological SPP1 assessment |
| Western blotting | 8 matched HCC and adjacent tissues | 8 patients | 16 tissues | SPP1 protein validation |
| ELISA | 58 patients with HCC and 36 healthy controls | 94 participants | 94 serum samples | OPN/AFP and clinical analyses |
