# Supplementary material for: SPP1 promotes anoikis resistance and metastatic phenotypes in hepatocellular carcinoma through PI3K-sensitive signaling
Source: Front Immunol. 2026 Oct 20;17:1980175. doi: 10.3389/fimmu.2026.1980175 (PMC13727754; doi:10.3389/fimmu.2026.1980175)
Supplement: Supplementary file 2 [file Table2.docx]

**Supplementary Table S2. Primer sequences used for qRT-PCR**

Primer sequences are shown in the 5′–3′ direction. ACTB, beta-actin; GAPDH, glyceraldehyde-3-phosphate dehydrogenase; qRT-PCR, quantitative reverse-transcription polymerase chain reaction.

| **Target** | **Primer** | **Sequence (5′–3′)** |
| --- | --- | --- |
| SPP1 | Forward | CGAGGTGATAGTGTGGTTTATGG |
| SPP1 | Reverse | GCACCATTCAACTCCTCGCTTTC |
| GAPDH | Forward | TGTTGCCATCAATGACCCCTT |
| GAPDH | Reverse | CTCCACGACGTACTCAGCG |
| ACTB | Forward | CATGTACGTTGCTATCCAGGC |
| ACTB | Reverse | CTCCTTAATGTCACGCACGAT |
